# Congenital heart disease in the Niger Delta region of Nigeria: a four-year prospective echocardiographic analysis

**DOI:** 10.5830/CVJA-2014-055

**Published:** 2014

**Authors:** BE Otaigbe, PN Tabansi

**Affiliations:** Department of Paediatrics, University of Port Harcourt Teaching Hospital, Port Harcourt Rivers State, Nigeria; Department of Paediatrics, University of Port Harcourt Teaching Hospital, Port Harcourt Rivers State, Nigeria

**Keywords:** congenital heart disease, high prevalence, Niger Delta, oil spillage

## Abstract

**Introduction:**

Echocardiographic evaluation remains the gold standard for the diagnosis of structural cardiac disease. No previous prospective studies have been done on the prevalence of congenital heart disease (CHD) in the Niger Delta area. This study was done to determine the frequency and pattern of congenital heart disease, using echocardiography as a diagnostic tool.

**Methods:**

All patients presenting to the Paediatric Cardiology clinics of two centres, the University of Port Harcourt Teaching Hospital and the Paediatric Care Hospital between April 2009 and March 2013, were recruited and all had echocardiography performed.

**Results:**

Prevalence of CHD in this study was 14.4 per 1 000 children; 277 (83.4%) of the patients had acyanotic CHD and 55 (16.6%) had cyanotic CHD. Ventricular septal defect and tetralogy of Fallot were the commonest acyanotic and cyanotic heart defects, respectively

**Conclusion:**

The high prevalence of CHD in this study is the highest in the country and Africa, and may be attributable to the increased oil spillage and gas flaring from petroleum exploitation in this region.

## Abstract

Congenital heart disease (CHD) affects approximately eight per 1 000 live births in the general population, making it one of the most common classes of birth defect.^1^ CHD is defined as an abnormality in the cardio-circulatory structure or function, which is present at birth, even if it is discovered later.^2^

More children die from CHD each year than are diagnosed with cancer.^3^ A large number of these children are in the developing countries. They are often repeatedly admitted and treated for recurrent chest infections and failure to thrive due to ignorance of the attending health caregiver, poor diagnostic tools, poor referral systems, and lack of skilled personnel. These lead to late diagnosis and increased mortality rates.

CHD can be life threatening in early childhood, and children born with severe forms are at approximately 12 times higher risk of mortality in the first year of life, especially if they are missed in the neonatal period.^4^ About two to three in 1 000 newborns with heart disease will be symptomatic in the first year of life, diagnosis is established by one week in 40–50%, and by one month of age in 50–60% of patients.^5^

The incidence of CHD in Nigeria 30 years ago was 3.5 per 1 000 population.^6^ This has increased in recent studies to 4.6/1 000 in the southern,^7^ and 9.3/1 000 in the northern^8^ parts of Nigeria, apparently due to increased diagnostic facilities and more trained paediatric cardiologists in the country.

Echocardiographic evaluation remains the gold standard for the diagnosis of structural cardiac disease.^7^ Paediatric echocardiography has not been widely available in Nigeria as there are few paediatric cardiologists and most of the available echocardiography machines have no paediatric probes. Prior to the procurement of an echocardiography machine with paediatric probes in the reporting echocardiographic centre, history, clinical diagnosis and chest radiography were used as tools for provisional diagnosis of CHD.^9^

There has been no previous report on the incidence of CHD in the Niger Delta region of Nigeria. Previous studies on CHD from Nigeria have been retrospective studies and none from the Niger Delta region of Nigeria, where there have been claims of increasing risks to maternal and child health due to environmental degradation and industrial pollution secondary to petroleum mining and gas flaring in this region. This study was done to determine the frequency and pattern of CHD, using echocardiography as a diagnostic tool.

## Methods

This was a prospective study of all patients presenting to the paediatric cardiology clinics of two centres, the University of Port Harcourt Teaching Hospital and the Paediatric Care Hospital, having been referred to the clinics or seen in the wards between April 2009 and March 2013. All the patients enrolled were fully examined by at least one of the two paediatric cardiologists and further evaluated with chest radiographs, electrocardiograms (ECG) and an echocardiogram (echo).

The chest radiographs were read separately by the cardiologist and radiologists and conflicting reports were discussed. The ECG was performed by a technician using a Schiller AT-1 Smart Print machine, standardised at a paper speed of 25 mm/s. Echo diagnosis of all patients was done using Sonosite Micromaxx and Sonosite Edge machines, available only at the Paediatric Care Hospital. Each patient was recruited with a proforma, which contained records of name, age, gender, weight, indication for echocardiography, chest X-ray and ECG findings, and echocardiographic parameters, diagnosis, comments and outcome.

## Statistical analysis

Data were computed and analysed using Epi version 6.02. For the purpose of this report, only the echo findings were included in the analysis.

## Results

During the period of four years, 23 124 children presented at the Paediatric Department of the two centres. Of these, 440 children were referred for echocardiographic evaluation. Only 356 (81%) had echo done, out of whom 24 were found to have structurally normal hearts. Therefore 332 patients with cardiac anomalies were analysed. Prevalence of CHD in this hospital-based study was 14.4 per 1 000 children.

Of those analysed, there were 174 males (52.4%) and 158 females (47.6%) in a ratio of 1:1. The ages ranged from 0.25 to 180 months with a mean of 26.1 months. Thirty (9%) of the children were aged one month or less, 62% were between one month and one year, 24.4% between one and five years, and 13.7% above five years of age.

The commonest indications for an echo were murmur in 36% (of which 6% were incidental murmurs), fast breathing in 19.8%, failure to thrive in 11% and cyanosis in 9.9%. Others included features of dysmorphism and easy fatigability (Table 1). Thirty-six (11%) of the patients had features suggestive of Down syndrome (DS), while one patient had dysmorphism suggestive of William’s syndrome.^9^ Of the 36 patients with DS, over 60% had multiple cardiac defects, with ventricular septal defects (VSD)/patent ductus arteriosus (PDA) and VSD/atrial septal defect (ASD) accounting for 50%. Nine patients had congenital *Rubella* syndrome, with PDA accounting for over 50% of the cardiac defects.

**Table 1:** Indications for echocardiography

*Sign/symptom*	*Number of patients*	*% of patients*
Fast breathing	64	19.2
Failure to thrive	36	10.8
Murmur	83	25
Dysmorphism	36	10.8
Cyanosis	32	9.6
Recurrent pneumonia	13	3.9
Recurrent cough	16	4.8
Easy fatigability	23	6.9
Chest pain	6	1.8
Cardiomegaly on chest X-ray	6	1.8
Palpitation	5	1.5
Fainting attacks	6	1.8
Heart failure	21	6.33
Pre-term low birth weight	7	2.1

Two hundred and seventy-seven (83.4%) of the patients had acyanotic CHD and 55 (16.6%) had cyanotic CHD. In the acyanotic group, there were 149 males (53.8) and 128 females (46.2%), with no statistically significant difference, while there were 25 males (45.5%) and 30 females (54.5%) in the cyanotic group.

In the acyanotic group, solitary VSDs accounted for 32.5% of cases, solitary PDAs for 17.3% and solitary atrial septal defect (ASD) for 8.3%. Atrio-ventricular canal defect (AVCD) was seen in eight (2.9%) (Table 2). Congenital dilated cardiomyopathy was seen in two patients, one of whom had lost two older siblings in infancy with similar conditions. Of the two patients with hypertrophic cardiomyopathy, one was a macrosomic infant of a diabetic mother, who had spontaneous resolution of the hypertrophy by the fourth month. The other was an infant of a non-diabetic mother, who was managed in a peripheral hospital and died after two months from recurrent heart failure.

**Table 2:** Solitary acyanotic CHD types and frequency of occurrence

*Type of acyanotic CHD*	*No of patients*	*% of acyanotic CHD (n = 277)*	*% of total CHD (n = 332)*
Ventricular septal defect	90	32.5	27.1
Patent ductus arteriosus	48	17.3	14.5
Atrial septal defect	23	8.3	2.5
Pulmonary stenosis	3	1.1	0.9
Atrio-ventricular canal defect	8	2.9	2.4
Congenital dilated cardiomyopathy	2	3.6	0.6
Congenital hypertrophic cardiomyopathy	2	3.6	0.6

In the cyanotic group, transposition of the great arteries (TGA) was seen in 12 patients, and tetralogy of Fallot (TOF) in 28 patients, two of whom had associated ASD (pentalogy of Fallot). Truncus arteriosus was seen in three patients (Table 3). Table 4 shows the number of children with multiple congenital heart diseases.

**Table 3:** Cyanotic CHD types and frequency of occurrence

*Type of cyanotic CHD*	*No of patients*	*% of cyanotic CHD (n = 55)*	*% of total CHD (n = 332)*
Tetralogy of Fallot	28	50.9	8.4
Transposition of the great arteries	12	21.9	3.6
Tricuspid atresia	3	5.5	0.9
Truncus arteriosus	3	5.5	0.9
Double-outlet right ventricle	3	5.5	0.9
Ebstein’s anomaly	3	5.5	0.9
Hypoplastic left heart syndrome	2	3.6	0.6
Partial anomalous pulmonary venous connection	1	1.8	0.3

**Table 4:** Multiple CHD and frequency of occurrence

*Type of CHD*	*Number*	*% acyanotic CHD (n = 277)*	*% total CHD (n = 332)*
VSD/PDA	53	19.1	16
VSD/ASD	6	2.2	1.8
ASD/PDA	16	5.8	4.8
ASD/VSD/PDA	12	4.3	3.6
ASD/PAPVC	2	0.7	0.6
AVCD/PDA	2	0.7	0.6

VSD, ventricular septal defect; PDA, patent ductus arteriosus; ASD, atrial septal defect; PAPVC, partial anomalous pulmonary venous connection; AVCD, atrio-ventricular canal defect.

Of the children studied, 24 (7.2%) have had successful surgeries in India, one in Ghana, one in South Africa and three in the United State of America. Two children with atrioventricular septal defect and pulmonary hypertension, who had Eisenmenger syndrome, went to India and were confirmed inoperable after cardiac cathetherisation.

One hundred and thirty-three (40%) of these children have had at least one hospital admission, and 10 (3%) have died. Four died in hospital while the other six were confirmed dead by telephone calls from parents. Of those who died in hospital, two had tetralogy of Fallot with cerebrovascular accident and died after partial exchange of blood for packed cell volume of 88 and 76%, respectively. The other two died of intractable heart failure.

## Discussion

The prevalence of CHD in this prospective, hospital-based study of 14.4 per 1 000 is alarmingly higher than studies from other parts of Nigeria, where it was 9.3,^8^ and 4.6/1 000,^7^ Egypt was 1.01,^10^ India was 10.5,^11^ the United State of America was 6.5,^12^ Norway was 10.6,^13^ and Austria was 6.9/1 000.^14^ It is comparable with other studies from Qatar where prevalence was 12.25,^15^ and Australia was 17.5/1 000.^16^

This very high figure is most likely due to environmental factors. Port Harcourt is in Rivers state in the Niger Delta region, an oil-rich city in the south–south geographical zone of Nigeria where crude oil exploration is rampant, and oil spillage from petroleum exploration commonly affects water quality and terrestrial fauna. Gas flaring constitutes a toxic threat to inhabitants of these areas. Heavy hydrocarbons that cannot be carried into the atmosphere fall back and become inhaled, while others get attached to vegetables grown for consumption. Over time, this may be toxic to the body or cause congenital malformations in babies born in the area.^17^ Toxic agents may induce malformation in the foetus during the early weeks of organogenesis.

As with other studies done in Nigeria and other parts of the world, VSD was the commonest acyanotic CHD seen in this study, with a frequency of almost half of the acyanotic CHD (47. 3%); 39.5% of the CHD we saw was similar to that seen in Saudi Arabia,^18^ Mysore,^12^ and Qatar.^15^ The prevalence of VSD was higher than previously reported in studies in Port Harcourt Teaching Hospital, which was 34.1%,^9^ 30.3% in Kano,^8^ 32.3% in the UK,^19^ 32.1% in the USA,^13^ and 35.6% in Egypt,^10^ but less than the 55.3% reported in Benin.^7^

TOF was the commonest cyanotic CHD, similar to studies in Nigeria,^7^-^9^ and worldwide.^10^-^16^ That TGA ranks second may be due to early mortality of the children. The two cases of dextrocardia had rare presentations of situs solitus with no structural heart defect,^20^ dextrocardia, and situs inversus with multiple CHD.^21^

Despite murmurs being the commonest indication for requesting an echo, there are still many patients presenting late to hospital, despite having been seen by numerous doctors managing them for recurrent bronchopneumonia and tuberculosis. This is due to the inability of attending junior medical staff to identify a murmur on auscultation. This is further highlighted in this study with only 9% of the patients reporting to hospital within the first month of life.

Increased risks of structural birth defects and chromosomal abnormalities have been reported to be due to air pollution and proximity to environmental waste. The findings of a large number of multiple congenital heart defects in these children is worrisome and may be related to the teratogenic effect of gas and oil spillage in the Niger Delta.

In this study, although no direct efforts were made to get information about the place of residence of the parents, a cursory review of the addresses showed that 28 (8.4%) of the parents lived close to areas where gas is flared, nine (0.23%) near telecommunication masts and 12 (0.04%) of the mothers, when asked what routine antenatal drugs were ingested, had mentioned a drug called Pregnacare. This contains multivitamins as supplements, including omega-3, folic acid, iron and vitamin B_12_.

These incidental findings have prompted an ongoing study emphasing description of location and review of drugs ingested by mothers of all children presenting with congenital heart disease in our centres. Hypervitaminosis, potentially teratogenic fumes and ionising radiation are being postulated as contributing factors to this high prevalence of CHD.

It may be worth mentioning that six of the infants with acyanotic heart disease were products of *in vitro* fertilisation (IVF). All six had multiple CHD and were all of multiple gestation. One patient, one of a set of triplets, had hypoplastic left heart syndrome (HLHS) and died within one week of diagnosis, at two weeks of life. The other siblings had structurally normal hearts. Alhough not statistically proven in this study as no comparison was made with children of parents who conceived naturally, the high risk of CHD in IVF patients has been previously documented.^22^,^23^ This has also prompted an ongoing prospective research on cardiac anomalies among patients delivered by IVF in the Niger Delta area.

These results may be the tip of the iceberg, as few of these cardiac patients present to hospital for diagnosis and fewer still can afford the cost of an echocardiograph. Most of the patients are still being managed by pharmacists and herbalists, mostly due to poverty, ignorance and poor access to proper medical care.

## Conclusion

The high prevalence rate of CHD reported in this study area, which has many environmental risks for CHD, is worrying. With the increasing availability of echocardiography and paediatric cardiologists in the region, and increased awareness, more cases are likely to be detected in future. There is an urgent need to assess and confirm the impact and causal relationship of oil spillage, gas flaring, use of the drug Pregnacare, and residence close to telecommunication masts in the Niger Delta, to make a genuine case for the prevention and reduction in the prevalence of CHD in this region. We emphasise the need to regulate the deleterious activities of oil companies in the Niger Delta, and establish cardiac centres in our country for cheaper and more easily available diagnostic tools and early surgery to improve outcomes.
